# Analysis of protected species observer data: Strengths, weaknesses, and application in the assessment of marine mammal responses to seismic surveys in the northern Gulf of Mexico 2002–2015

**DOI:** 10.1371/journal.pone.0300658

**Published:** 2024-03-21

**Authors:** Mary Jo Barkaszi, Christopher J. Kelly

**Affiliations:** CSA Ocean Sciences Inc., Okeechobee, FL, United States of America; Universidad de Cadiz Facultad de Ciencias del Mar y Ambientales, SPAIN

## Abstract

Visual observation data collected by protected species observers (PSOs) is required per regulations stipulated in Notices to Lessees (NTLs) and geophysical survey Permits (Form BOEM-0328) issued to seismic operators in the Gulf of Mexico (GOM). Here, data collected by certified and trained PSOs during seismic surveys conducted between 2002–2015 were compiled and analyzed to assess utility in assessing marine mammal responses to seismic noise and effectiveness of required mitigation measures. A total of 3,886 agency-required bi-weekly PSO Effort and Sightings reports were analyzed comprising 598,319 hours of PSO visual effort and 15,117 visual sighting records of marine mammals. The observed closest point of approach (CPA) distance was statistically compared across five species groupings for four airgun activity levels (full, minimum source, ramp up, silent). Whale and dolphin detections were significantly farther from airgun array locations during full power operations versus silence, indicating some avoidance response to full-power operations. Dolphin CPA distances were also significantly farther from airguns operating at minimum source than silence. Blackfish were observed significantly farther from the airgun array during ramp up versus both full and minimum source activities. Blackfish were observed significantly closer to the airgun array during silent activities versus at full, minimum source, and ramp up activities. Beaked whales had the largest mean CPA for detection distance compared to all other species groups. Detection distances for beaked whales were not significantly differences between full and silent operations; however, the sample size was very low. Overall results are consistent with other studies indicating that marine mammals may avoid exposure to airgun sounds based on observed distance from the seismic source during specified source activities. There was geographic variability in sighting rates associated with specific areas of interest within the GOM. This study demonstrates that agency required PSO reports provide a robust and useful data set applicable to impact assessments; management, policy and regulatory decision making; and qualitative input for regional scientific, stock assessment and abundance studies. However, several improvements in content and consistency would facilitate finer-scale analysis of some topics (e.g., effort associated with specific activities, observer biases, sound field estimation) and support statistical comparisons that could provide further insight into marine mammal responses and mitigation efficacy.

## Introduction

### Purpose of this study

United States Federal regulations require placing protected species observers (PSOs) on board seismic survey platforms in the Gulf of Mexico (GOM). The PSOs are responsible for implementing mitigation measures designed to minimize impacts from the interaction of protected species with sound-producing marine activities. Prescribed reports, defined by government regulations, are produced by the PSOs. Over time in the U.S. Gulf of Mexico (GOM) these reports have resulted in a large volume of data being amassed by the Bureau of Ocean Energy Management (BOEM). Here, we conducted a retrospective analysis of those data to 1) determine their potential for providing guidance regarding protected species management and mitigation during deep-penetration seismic surveys, and 2) assess the utility and betterment of marine species data collection under the current regulatory requirements.

### Seismic survey sound

Geophysical surveys use seismic and non-seismic sound sources to investigate the shallow seafloor for archaeologic, hazard detection, and engineering site work, or for deeper illumination (several kilometers below the seafloor) of seabed geologic structures, usually in search of potential oil and gas deposits [1]. Seismic surveys conducted for oil and gas exploration in the GOM typically use airgun sources in conjunction with towed or ocean bottom-placed receivers, called hydrophones and geophones, respectively [1]. Airguns produce low-frequency sound by releasing controlled volumes of high-pressure air into the water, sending an acoustic pulse into the seabed [1, 2]. The return of the acoustic pulse is recorded by the receivers as each pulse is reflected by the various layers in the seafloor. The recorded signals are then analyzed and processed to yield information on the subsea geological structures. Airgun source frequencies typically range from approximately 10 to 2,000 Hz, with most energy below 1,000 Hz [1–4]. The source volume of air generated by an individual airgun can range from less than 5 in^3^ to over 2,000 in^3^ with acoustic impulse amplitudes correlating to source volumes [2, 3]. Total source volume of a multi-airgun array may reach 8,460 in^3^ with source levels having a zero-to-peak sound pressure approaching 250 dB re 1 μPa m [1]. Source levels produced by seismic airgun arrays are of sufficient amplitude and frequency to meet physiological and/or behavioral acoustic threshold criteria for influencing marine mammal and sea turtle species protected under the Marine Mammal Protection Act (MMPA) and Endangered Species Act (ESA) [5–7]. Other effects from underwater sound (e.g., masking) may be realized by marine mammals, sea turtles, and other species; however, the data collected for this analysis correlated to specific regulatory roles.

### Protected species

Twenty-two marine mammal species inhabit the northern GOM [8, 9]. These marine mammals include one species of baleen whale, the Rice’s whale (*Balaenoptera ricei*), which is primarily found along the bathymetric feature known as De Soto Canyon off the northwest Florida coast; one sirenian species, the West Indian manatee (*Trichechus manatus*), which is typically found in coastal and inshore waters; and 20 species of more broadly distributed toothed whales and dolphins (suborder: Odontoceti). All the marine mammals are protected under the MMPA. The sperm whale, *Physeter macrocephalus*, and Rice’s whale, *Balaenoptera ricei*, are also listed as endangered under the ESA. At the time of data collection and reporting reflected in this paper, Rice’s whale was still classified as Bryde’s whale (*Balaenoptera edeni*) and was not listed under the ESA [9]. This paper reflects the updated taxonomy and listing status for Rice’s whale; all baleen whale records from PSO reports are assumed to be Rice’s whales [8, 9]. The sperm whale is the most abundant large whale in the GOM and is widely distributed across slope and canyon habitats [9]. In addition to marine mammals, there are also five sea turtle species that inhabit the northern GOM, all of which are listed as endangered or threatened under the ESA and were therefore part of the PSO reporting requirements. Sea turtle analyses conducted as part of the corresponding BOEM report [10] are not included in this paper.

### Regulatory roles

BOEM and the Bureau of Safety and Environmental Enforcement (BSEE) are responsible for managing impacts of anthropogenic underwater sound on protected species in the northern GOM to comply with the MMPA, ESA, and National Environmental Policy Act (NEPA) per the Outer Continental Shelf Lands Act (OSCLA). BOEM and BSEE convey regulations to offshore operators via a Notice to Lessees (NTL) which mandates operating procedures necessary to comply with the ESA and MMPA. The data collected that were analyzed in the paper were collected under several NTLs that have since expired (e.g., NTL 2007-G02; Joint NTL No 2012-G02; BOEM NTL No. 2016-G02). Prior to 2013, requirements outlined in NTLs applied to all seismic survey activities conducted under lease terms in northern GOM waters deeper than 200 m. That water depth was decreased to 100 m because of the 2013 Settlement Agreement between the Department of the Interior and the Natural Resources Defense Council, et al. (Civil Action No. 2:10-cv-01882). NTL requirements also pertain to seismic survey activities within all waters, regardless of water depth, east of 88.0° W longitude, a region offshore Florida commonly referred to as the Eastern Planning Area.

Mitigation measures designed to protect marine mammals from sound exposures during seismic surveys are defined within the NTL and in requirements associated with the 2013 Settlement Agreement. Additionally, permit-specific conditions may be issued in the operator’s geological and geophysical (G&G) permit. Typical mitigation measures applied during a seismic exploration survey include 1) a visually and/or acoustically monitored (marine species) exclusion zone, 2) an exclusion zone clearance period prior to the initiation of airgun activity, 3) a gradual ramp up of the airgun output levels upon initiation of seismic operations, 4) operation of a minimum acoustic output source to maintain an ensonified field around the sound source to prevent protected species from entering the exclusion zone during lulls in survey activity, and 5) shutting down or delaying the start of airgun operations if an animal enters the exclusion zone around the sound source [11–13]. However, few studies [14, 15] have systematically addressed the effectiveness of these mitigation measures to minimize injury-level sound exposures to protected species during seismic surveys.

Required PSO and seismic survey mitigation measures have been in place in the GOM since 2002 with little variation in the subsequent NTL mitigation and monitoring requirements. The 2013 Settlement Agreement required additional mitigation in some areas of the GOM and individual permit conditions may introduce additional requirements. Compliance with NTLs, the 2013 Settlement Agreement, and permit conditions is required of operators to conduct seismic survey operations and lease activities in the northern GOM. The mitigation and monitoring program implemented as part of these conditions requires visual observations, and in some cases passive acoustic monitoring (PAM), to be conducted and reported by trained personnel (PSOs) onboard all vessels and platforms operating airgun sources within specified water depths. Compliance oversight for these measures is provided through the PSO program to the Protected Species Program in BSEE by report submission every two weeks (bi-weekly). Each bi-weekly report compiles seismic survey operations and visual and acoustic observational data for that two-week time period as part of the NTL and permit condition requirements issued to seismic operators in the northern GOM. Under the NTL reporting requirements there are no comprehensive or final summary reports associated with individual surveys or G&G permits; only the bi-weekly submission.

### Retrospective analysis

We summarized bi-weekly PSO data submitted to BSEE from seismic surveys conducted in the northern GOM, 2002–2015. The purpose of this study was threefold: 1) extract relevant information from PSO data to provide regulators with a means to assess and improve the level of compliance reporting, 2) evaluate potential impacts of seismic operations on protected species, and 3) assess the effectiveness of mitigation measures designed to protect marine mammal species from injury-level sound exposures. Notably, though the underlying PSO mitigation data collected for this 14-year period was extensive, data collection was designed and intended for reporting operator compliance with the narrow NTL-specific requirements. Therefore, data did not conform to standard methodologies used for other purposes, such as species density estimation or behavioral response studies. PSO report data were first examined for adequate data structure, replication and information content to provide potential insight on ways to expand or fine-tune seismic mitigation measures. The most consistently reported data suitable to address the study purpose pertained to the species’ closest point of approach (CPA) distance to the sound source location at various airgun operational levels.

We evaluated the structure and composition of the PSO reports to assess adequacy for statistical analyses. For those data elements considered adequate, we assessed whether the CPA varied among various categories of marine mammals defined by taxonomic and sound frequency hearing groups, for each of four airgun activity levels (full, minimum source, ramp up, silent). CPA distances at which animals were detected at various output levels of airguns is used here as a proxy for potential avoidance or non-avoidance behavior, as done in other studies (e.g., Stone [13]; Nowacek et al., [16]).

## Methods

### Foundation data

During seismic surveys, at least three trained PSOs are required by BSEE to conduct visual observation during daylight hours on survey vessels and platforms operating an airgun source. The three PSOs are required to conduct watches on a rotational shift using two PSOs on concurrent watch and one PSO on rest with no single PSO exceeding a watch shift of 4 hours without a rest shift. Operators can either use trained third-party PSOs contracted specifically for these surveys, or trained crew members whose shipboard responsibilities and rest periods do not temporally overlap with their assigned PSO shift. The NTLs requires that three separate data reports are filled in daily, and submitted bi-weekly by PSOs to BSEE, during a survey: the Effort Report, the Sighting Report, and the Survey Report (the latter excluded from analysis conducted for this paper) (Table 1) (BOEM NTL No. 2016-G02).

**Table 1:** Information required for the three data reports for mitigation reporting during seismic survey operations in the northern Gulf of Mexico as described in BOEM NTL 2016-G02.

Effort Report	Survey Report	Sighting Report
• Vessel name • Date • Survey type (e.g., high resolution, VSP, 2D, 3D) • BOEM permit number or Plan Control Number/OCS Lease Number • Observers’ names and affiliations • Time, latitude, and longitude when daily survey visual began and ended. • Average environmental conditions while on visual survey ○ Wind speed/direction ○ Sea state (glassy, slight, choppy, rough, or Beaufort scale) ○ Swell (low medium, high, or swell height in meters) ○ Overall visibility (poor, moderate, good)	Vessel name • Date • Survey type (e.g., site, 3D, 4D) • BOEM permit number or Plan Control Number/OSC Lease Number • Time pre-ramp up survey begins. • Marine mammals and sea turtles seen during pre-ramp up survey. • Time ramp up begins. • Whales seen during ramp up. • Time airgun array is operating at the desired intensity. • Marine mammals and sea turtles were seen during survey. • Any action taken (i.e., survey delayed, guns shut down) if whales were seen. • Reason that whales might not have been seen (e.g., swell, glare, fog) • Time airgun array stops firing	Vessel name • Date • Survey type (e.g., site, 3D, 4D) • BOEM permit number or Plan Control Number/OCS Lease Number • Time • Watch status. • Observer or person who made sighting. • Latitude and longitude of vessel • Heading of vessel • Bearing and estimated range to animal(s) at first sighting • Water depth (meters) • Species (or identification to lowest possible taxonomic level) • Certainty of identification (sure, most likely, best guess) • Total number of animals • Number of juveniles • Description of animal (as many distinguishing features as possible) • Direction of animal’s travel—compass direction • Direction of animal’s travel—relative to vessel • Behavior (as explicit and detailed as possible; note any observed changes in behavior) • Activity of vessel • Airguns firing (yes or no) • Closest distance (meters) to animals from center of airgun or airgun array (whether firing or not)

	
	
	
	
	
	
	
	
	

Effort Reports summarize observer monitoring times, locations, and environmental conditions. Survey Reports provide information pertaining to the ramp up protocols and clearance of the exclusion zone prior to the start of airgun deployment and the general operational parameters observed during operations. Sighting Reports provide specific information pertaining to each sighting of a protected species during seismic survey operations including vessel and seismic activity, species identification, animal behavior, and as relevant, acoustic detections when PAM is used. Effort and Survey Reports are compiled daily, while Sighting Reports are compiled for each individual observation of a protected species during seismic survey operations. All three reports are submitted to BSEE on the 1st and 15th of each month during which a seismic survey is conducted. In the GOM, standard practice is for these reports to first go through a PSO/PAM provider company supervisor who reviews the reports and submits them to BSEE. Thus, shipboard PSOs typically do not submit reports directly to BSEE.

The implementation of PAM as a monitoring method aboard seismic surveys has evolved over the years. Although considered an optional measure in the BOEM NTLs, PAM was required in some permit conditions after 2010 and was stipulated in the 2013 settlement agreement for all deep-penetration surveys conducted in water depths over 100 m. PAM has generally become a widely accepted monitoring method for marine mammal mitigation on seismic vessels [11, 17] due, in part, to the continued improvement of PAM systems and its demonstrated efficacy in both research and other monitoring applications [17–20]. The efficacy of towed PAM systems is limited for low frequency species and very little is published about quantifying PAM’s efficacy as a seismic mitigation measure [17, 21]. Only recently have standards been developed regarding acceptable equipment, monitoring methods, and reporting for towed PAM systems [17, 21]. PAM systems deployed on seismic programs are inherently susceptible to high noise conditions which can decrease a PAM system’s or PAM operator’s ability to effectively detect, classify, and localize marine mammal species [17, 21]. We did not analyze PAM data herein due to the lack of accurate distance determination and absence of error reporting or detection distance assessment in all PAM reports.

#### Data synthesis

We applied a quality control process to assess the usability of the data. The bi-weekly report data were provided to the authors (through BSEE contract) in the original format provided to BSEE by PSO providers, which consisted mainly of portable document format (pdf) files. Data from 2002–2008 were available in Microsoft Excel (Excel) format but only the last 16 months (2014–2015) of bi-weekly reports were submitted to BSEE in Excel format. Therefore, transcription of data from the bi-weekly pdf PSO reports between 2009 and 2014 to an Excel spreadsheet was required to build a standard query language (SQL) database in Microsoft Access for the entire data set. Although most reports contained all three forms (Effort, Sighting, Survey), data entry errors were common in all the forms (e.g., incorrect time or latitude/longitude formats, blank data cells). Such errors could not be corrected and were thus excluded from our analysis. In most cases, these errors did not render the entire form or report unusable and only excluded sections of data within a single bi-weekly report. These data inconsistencies arise in part because no standard data forms are required by BSEE; however, most PSOs used some altered version of data forms digitally available from the United Kingdom’s Joint Nature Conservation Committee (JNCC). We used these form headings as a template to build the SQL database because the data headings corresponded with data headings provided in the majority of PSO forms. Location and time data recorded by PSOs displayed the greatest inconsistencies in format and spreadsheet errors (e.g., #VALUE!, #####, #REF!); none of which could be recovered because the submitted data was in pdf format and manipulation in Microsoft Excel or Access could not be performed. The pdf forms were submitted to BSEE with these errors in place. For our analysis, data entry personnel copied the exact data found within the reports into the Excel spreadsheet except the latitude and longitude which were converted to a common, decimal degree format to support spatial analysis. Other fields contained various inputs such as symbols, words, numbers, or a combination thereof. To avoid potential bias, unintentional changing of data, or misinterpretation of data during the data entry phase, the other fields were copied over to the Excel database exactly as they were provided to BSEE. For example, some start times for monitoring indicated “continuous monitoring” instead of a specific time, thus the words “continuous monitoring” were entered into the database instead of forensically interpreting the potential times that monitoring may have begun. These format inconsistencies and lack of standardized, digital data entry resulted in manual entry and several layers of quality assurance checks versus automation.

We evaluated the Microsoft Access database and assessed the quality and completeness of each record. We also determined the suitability of each record for statistical analysis by determining its independence from other observations, its variable type (i.e., categorical, continuous), and likelihood of accuracy relative to both itself (i.e., compared with other metadata for that observation) and other observations (i.e., if it was a probable outlier).

Previous studies have shown that as the distance increases, shipboard PSOs become less effective at detecting animals and less accurate in distance estimations, and the accuracy of the recorded observation consequently becomes less reliable [22]. Some reported distances of animals by PSOs were as far away as 7 km (without big eye binoculars), a distance at which the accuracy of an observation is subject to great uncertainty [22–24]. A truncation distance was therefore used in our analysis to reduce bias in the dataset associated with observations of potential questionable validity or relevance. Truncation distance to analyze PSO visual distance data has been used previously in survey data described within the marine mammal population survey literature. Barlow [23] used truncation distances ranging from 4.0 km for beaked whales and *Kogia* spp. to 5.5 km for dolphins and large whales during systematic research surveys. Williams [25] demonstrated that detection probability and measurement accuracy dropped off significantly at 2 km for large whale species using the naked eye and hand-held binoculars and that measurement error between observers introduced substantial bias even when using reticle binoculars. Barlow and Gisner [22] estimated that the likelihood of detecting beaked whales on mitigation surveys was 24 to 48 times lower than the detection probability on research surveys; further, the probability of detecting beaked whales with 7x binoculars drops to zero at approximately 1 km from a vessel. Stone [13] applied a qualitative criterion to observer data by assigning a higher level of data reliability to data recorded by “experienced” observers who were defined as those have a minimum of 20% of their detections more than 1 km away; however, given that we find that unskilled observers routinely recorded detections at this distance, we did not use that metric as a reliable source of data validation. In a data summary of PSO observations, Stone [13] computed median CPAs for 13 marine mammal species during airgun firing and airgun silence for different array types. The median CPAs ranged from <500 m for seals and common dolphins to 2 km for sperm whales and bottlenose dolphins and 3 km for minke whales.

Based on the above information, all whale and dolphin data were truncated at 2,500 m with the understanding that small whales (*Kogia* spp.) and beaked whales may be missed at these distances. However, given the preponderance of sperm whales sightings within this range and to provide for some error in distance estimation, we used this distance (2,500 m) as a cut-off for reliable sighting data. Further, we believed including distances greater than 2,500 m would introduce unnecessary bias into our analysis, resulting in a reduction in the confidence of the results.

Our overall analytical investigation was designed to determine statistical differences in the observations reported by the PSOs for each animal group to the four airgun activity levels. CPA distances to airgun sources operating at different power levels could indicate behavioral responses if those distances are significantly different between airgun power levels.

Data were separated out into five animal groups correlating to the two regulatory categories defined in the NTL (all whale species and all dolphin species) and three of the NMFS [5] auditory groups: low frequency (7 Hz – 35 kHz), mid frequency (150 Hz– 160 kHz), and high frequency (275 Hz– 160 kHz). NTL categories are based on phylogenetic classification of animals identified in regulatory mitigation requirements, while the NMFS auditory groupings are based on the accepted hearing frequency sensitivities of cetaceans [5]. However, the auditory groups may not capture the variability in animal response to sound because of the behaviorally and ecologically distinct species that are grouped in the mid-frequency category which is the primary auditory group the GOM. Given that the volume of PSO observations is dominated by only a few species, those species may drive the results of the entire mid-frequency group. Therefore, within the mid-frequency auditory grouping, several additional investigations were conducted to assess the responses of various behaviorally and ecologically similar (non-regulatory) groupings to airgun activity levels per Table 2 (NTL categories), Table 3 (auditory grouping), and Table 4 (mid-frequency faunal grouping).

**Table 2:** Categories of cetacean species as defined in the Notice to Lessees (NTL). The most commonly identified species by PSOs within each NTL category are shown in bold-faced type.

NTL Category	Species
All Whale Species	**Sperm whale, *Physeter macrocepahalus***
	Bryde’s whale, *Balaenoptera edeni*
	Pygmy sperm whale, *Kogia breviceps*
	Dwarf sperm whale, *Kogia sima*
	Blainville’s beaked whale, *Mesoplodon densirostris*
	Gervais’ beaked whale, *Mesoplodon europaeus*
	Cuvier’s beaked whale, *Ziphius cavirostris*
	Unidentified whale species
All Dolphin Species	Pygmy Killer Whale, *Feresa attenuata*
	Short-finned Pilot Whale, *Globicephala macrorhynchus*
	Risso’s Dolphin, *Grampus griseus*
	Fraser’s Dolphin, *Lagenodelphis hosei*
	Killer Whale, *Orcinus orca*
	Melon-headed Whale, *Peponocephala electra*
	False Killer Whale, *Pseudorca crassidens*
	**Pantropical Spotted Dolphin, *Stenella attenuata***
	Clymene Dolphin, *Stenella clymene*
	Striped Dolphin, *Stenella coerulealba*
	Atlantic Spotted Dolphin, *Stenella frontalis*
	Spinner Dolphin, *Stenella longirostris*
	Rough-toothed Dolphin, *Steno bredanensis*
	**Bottlenose Dophin, *Tursiops truncatus***
	Unidentified dolphin species

**Table 3:** List of cetacean species categorized for low, medium, and high frequency auditory groups based on regulatory definitions. The most commonly reported species within each auditory category are shown in bold-face type.

Auditory Groups	
Low Frequency (7 Hz to 35 kHz)	**Bryde’s whale, *Balaenoptera edeni***
Mid-Frequency (150 Hz to 160 kHz)	**Sperm whale, *Physeter macrocepahalus***
	Blainville’s beaked whale, *Mesoplodon densirostris*
	Gervais’ beaked whale, *Mesoplodon europaeus*
	Cuvier’s beaked whale, *Ziphius cavirostris*
	Pygmy Killer Whale, *Feresa attenuata*
	Short-finned Pilot Whale, *Globicephala macrorhynchus*
	Risso’s Dolphin, *Grampus griseus*
	Fraser’s Dolphin, *Lagenodelphis hosei*
	Killer Whale, *Orcinus orca*
	Melon-headed Whale, *Peponocephala electra*
	False Killer Whale, *Pseudorca crassidens*
	**Pantropical Spotted Dolphin, *Stenella attenuata***
	Clymene Dolphin, *Stenella clymene*
	Striped Dolphin, *Stenella coerulealba*
	Atlantic Spotted Dolphin, *Stenella frontalis*
	Spinner Dolphin, *Stenella longirostris*
	Rough-toothed Dolphin, *Steno bredanensis*
	**Bottlenose Dolphin, *Tursiops truncatus***
High Frequency (275 Hz to 160 kHz)	Pygmy sperm whale, *Kogia breviceps*
	Dwarf sperm whale, *Kogia sima*

**Table 4:** List of non-regulatory faunal subgroups analyzed within the mid-frequency auditory grouping.

Mid-Frequency Auditory Groups	
Sperm Whales	Sperm whale, *Physeter macrocephalus*
Bottlenose and Pantropical Spotted Dolphins	Pantropical Spotted Dolphin, *Stenella attenuata*
	Bottlenose Dolphin, *Tursiops truncatus*
Other Small Dolphins	Atlantic Spotted Dolphin, *Stenella frontalis*
	Clymene Dolphin, *Stenella clymene*
	Fraser’s Dolphin, *Lagenodelphis hosei*
	Rough-toothed Dolphin, *Steno bredanensis*
	Spinner Dolphin, *Stenella longirostris*
	Striped Dolphin, *Stenella coerulealba*
Blackfish	Short-finned Pilot Whale, *Globicephala macrorhynchus*
	Risso’s Dolphin, *Grampus griseus*
	Melon-headed Whale, *Peponocephala electra*
	False Killer Whale, *Pseudorca crassidens*
	Killer Whale, *Orcinus orca*
	Pygmy Killer Whale, *Feresa attenuata*
Beaked Whales	Blainville’s beaked whale, *Mesoplodon densirostris*
	Gervais’ beaked whale, *Mesoplodon europaeus*
	Cuvier’s beaked whale, *Ziphius cavirostris*

Within each animal grouping the dependent variable, CPA, was compared among the four independent variables corresponding to the airgun activity levels full power, minimum source, ramp up, and silent. Airguns silent does not equate to survey silence because vessel (or platform) operations will introduce sound into the water (e.g., engine noise, streamer and equipment towing noise); however, these sources were presumed to be relatively equal across airgun source levels and therefore are not considered as contributing more or less during any of the airgun operation. Full power was defined as airguns operating at power levels designed for geophysical data acquisition. Minimum source was the main airgun array or, a separate supplementary airgun was operating at a lower power level to maintain a minimum sound pressure level output of 160 dB re 1 μPa. Ramp up power involved gradual increase of the airgun output over 20 to 40 minutes. Airguns were off but may or may not have remained in the water during times categorized as airguns silent.

Each impulse produced by the airgun array propagates a distance proportional to the source level; therefore, each of these power levels represent a different potential for sound exposure or range at which a subsequent reaction by animals (e.g., aversion), may occur. The mitigation zone (500 m around the sound source) was established by BOEM as the estimated distance beyond which the impulse produced by a typical deep penetrating seismic source drops below the threshold for onset of auditory injury to marine mammals. Full power versus silent comparisons aid in parsing out potential reactions by animals specific to the airgun sound propagated through the water. Ramp up and minimum source levels are specific mitigation methods designed to protect animals from exposure to the highest source levels. Therefore, analysis of ramp up and minimum source provide comparisons of source output levels that fall between full power and silence giving some indication of responses to any airgun sources verses specific airgun source levels. More importantly, data from the ramp up and minimum source levels provided the opportunity to test whether these mitigation measures are effective in achieving the mitigation goals.

The same comparison was repeated for dolphin species found within 500 m of the airgun arrays to assess dolphin behavior in regard to various airgun activity levels within the defined exclusion zone. Sound levels within the exclusion zone are more intense relative to those outside of the exclusion zone and therefore may result in a different response by an animal. The additional analysis of dolphins observed within the 500-m exclusion zone is possible because a shutdown is not required for dolphins within this zone. In contrast, the airgun array must be shut down if a whale is detected within the 500-m exclusion zone.

Bow riding by dolphins creates a special circumstance for analysis because the bow riding behavior will influence the reported CPA and potential sound exposures. However, we were unable to account for any behavioral response differences between bow riding versus non-bow riding dolphins because this information was not consistently available or recorded in the data, we examined prior to 2014, when BOEM began requiring that bow riding information be collected. The corresponding BOEM studies report [10] provides discussion and descriptive analysis of the available bow-riding information that is not within the scope of this investigation.

Our data quality assessment process resulted in the analysis of 3,886 bi-weekly PSO reports submitted to BSEE during the 2002 – 2015 time period. This comprised 598,319 hours of PSO visual effort from the Effort Reports and 15,117 visual records extracted from the Sighting Reports. Table 5 provides a breakdown of the framework for each analysis. Table 6 summarizes the breakdown of metadata information relative to the truncation of NTL category data to within 2,500 m and 500 m of airgun arrays for variables investigated.

**Table 5:** Statistical investigations conducted on PSO observations^†^.

Animal Grouping	Animal Grouping Level	Truncation Distance (m)	Dependent Variable	Independent Variable (airgun activity)	Data Transformation	Analytical Investigation
NTL category	All Whale Species	2,500	CPA (m)	Full, Silent, Ramp up, Minimum Source	None	Differences in CPA of all whale species to various airgun activity levels
	All Dolphin Species^††^	2,500	CPA (m)	Full, Silent, Ramp up, Minimum Source	Square Root	Differences in CPA of all dolphin species to various airgun activity levels
		500	CPA (m)	Full, Silent, Ramp up, Minimum Source	None	Differences in CPA of all dolphin species observed within the exclusion zone to various airgun activity levels
Auditory grouping	Low	2,500	CPA (m)	Full, Silent	None	Differences in CPA of low frequency animals to various airgun activity levels
	Medium	2,500	CPA (m)	Full, Silent, Ramp up, Minimum Source	Square Root	Differences in CPA of mid-frequency animals to various airgun activity levels
	High	2,500	CPA (m)	Full, Silent	None	Differences in CPA of high frequency animals to various airgun activity levels
Mid-Frequency Auditory Group	Sperm Whales	2,500	CPA (m)	Full, Silent, Ramp up, Minimum Source	None	Differences in CPA of sperm whales to various airgun activity levels
	Bottlenose and Pantropical Spotted Dolphins	2,500	CPA (m)	Full, Silent, Ramp up, Minimum Source	Square Root	Differences in CPA of combined bottlenose and pantropical spotted dolphins to various airgun activity levels
Mid-frequency Auditory Group (cont’d)	Other Small Dolphins	2,500	CPA (m)	Full, Silent, Minimum Source	Square Root	Differences in CPA of all other combined dolphin species (Atlantic spotted, Clymene, Fraser’s, Rough-toothed, Spinner, and Striped dolphin) to various airgun activity levels
	Blackfish	2,500	CPA (m)	Full, Silent, Ramp up, Minimum Source	Square Root	Differences in CPA of blackfish to various airgun activity levels
	Beaked Whales	2,500	CPA (m)	Full, Silent	None	Differences in CPA of beaked whales to various airgun activity levels

†All statistical investigations were conducted first using a One-Way Analysis of Variance, and then a post-hoc Tukey Multiple Means comparison test, if applicable.

†† Species of dolphins exclusive of pantropical spotted, blackfish, and unidentified.

**Table 6:** Total number of observations (n) for the closest observed point of approach (CPA) for each NTL category.

Truncation Distance (m)	Parameter	NTL Category	Total PSO Observations (n)	Observations Removed (n)^1^		Total Observations Analyzed (n)
				n	%	
2,500	CPA	All Whale Species	2,999	708	23.6	2,291
		All Dolphin Species	8,623	285	3.3	8,338
500	CPA	All Whale Species	--	--	--	--
		All Dolphin Species	8,623	3,099	35.9	5,524

-- = data not applicable.

^1^ Observations removed (n and %) and total observations analyzed (n) after the data distance truncation.

#### Statistical analysis

Data were analyzed using SAS 9.2 Statistical software. To proceed with parametric analysis of the data, any violations of the assumptions of normality and homogeneity of variance (HOV) were investigated on the raw data. The assumption of normality was statistically (i.e., Kolmogorov-Smirnov) tested and graphically (i.e., box-plot, histogram, normal probability plot) examined for non-conformity of the sampling distribution. The assumption of HOV was also tested both statistically (i.e., Levene’s HOV Test) and graphically (i.e., plot of residuals versus predicted values). If it was found from the statistical tests or judged from the graphical analysis that raw data violated either of these assumptions then an appropriate transformation (i.e., square root) was applied to attempt to normalize data distribution and improve the similarity of variances. Square root data transformations were applied to the CPA of all dolphin species observations made within 2,500 m of airgun arrays. A square root data transformation was also applied to the mid-frequency auditory grouping, and certain faunal subgroups within the mid-frequency auditory grouping (Table 5). All other data sets were normally distributed. Table 5 provides a breakdown of the data transformation utilized for each data set.

Data for each investigation was analyzed using a One-Way Analysis of Variance (ANOVA) test by PROC MIXED to determine statistical differences of observations among the four airgun activity levels (full power, minimum source, ramp up, and silent) for the dependent variable (CPA) for each NTL or auditory category. Statistical differences among airgun activity levels within each NTL or auditory category were determined using a post-hoc Tukey Multiple Means Comparison Test.

## Results and discussion

### PSO data quality

As noted previously, data were collected primarily to determine seismic operator compliance and not under a statistically defined framework or necessarily in a manner conducive to statistical analysis. The original field reports represented a large amount of high-quality data; however, there are important overarching caveats that limit its utility for some analyses common to marine mammal surveys. For example:

1. When multiple vessels are operating simultaneously, detections are not coordinated between vessels. Each vessel is required, through the regulatory NTL, to establish its own exclusion zone and maintain its own data and reporting even when operating in conjunction with other survey vessels for the same survey. Therefore, multiple vessels that detect the same animals record these detections independently. This becomes an issue in particular for data collected after 2010 when the preponderance of surveys were multi-vessel [10]. Multi-vessel surveys comprised 70% of all PSO effort hours during 2010–2013 involving wide azimuth (WAZ) surveys that typically employed 2 or more concurrent source vessels. After 2013, most PSO effort involved full azimuth (FAZ) surveys that typically employed 3 or more concurrent source vessels. In addition to affecting data independence, the potential for duplicative sightings reported during multi-vessel surveys could result in behavioral differences in animals when encountering multiple source vessels, with variability from these encounters embedded in the variance of the CPA data.
2. Initial evaluation of the data revealed considerable variability in PSO watch methodology. Because observers must monitor compliance, standard survey methodologies are not employed, and surveyed areas are not equal. For example, observers often monitor the stern of the vessel where the airguns (and thus the exclusion zone) are located which may affect detection of animals that the vessel is approaching; observers may focus observations at different distances (e.g., one surveys 500m while others survey 2 km); observers’ fields of view for monitoring may or may not be contiguous (e.g., each observer does not watch a discrete section of the field of view); vessel speed and heading vary due to geophysical survey requirements but are not reliably recorded in the PSO data; and observers are not always stationed in the same location throughout a survey. Therefore, distance sampling analysis was not attempted because the associated assumptions were not consistently met.
3. Although sea state and visibility conditions are reported by PSOs, there was inconsistent recording of effort under each environmental condition across reports (i.e., some observers recorded visibility conditions whenever conditions changed, some recorded sporadically, some recorded hourly, some recorded at start and end of watch, etc.); therefore, analysis regarding species detection bias cannot be fully assessed.
4. At the time the data were collected under the NTL, PSOs were required to have successfully completed an approved training course but were not required to have any previous marine mammal survey experience or prior knowledge of marine mammal species or behavior. Therefore, consistency in observation interpretation and recording vary as a function of experience.
5. Consistent methodologies for range finding were not applied, or at least not reported. Distances using reticle binoculars were common, but calibration of the binoculars was rarely provided; therefore, precise ranging was not assumed in the data.

Input inconsistencies or errors in the PSO reports did not render data unusable and we compiled a highly robust data set for analysis from the large volume of PSO data; however, the detail to which some of the analyses can be made was affected. For example, species detection rates could be calculated based on the overall observer effort recorded, but detection rates specific to airgun power or environmental conditions (i.e., detection rates during full power operations only; or detection rates during a specific sea state) could not be determined beyond descriptive statistics.

### Protected species composition

Table 7 summarizes the 18 species, mean group size, visual detection rate, mean water depth, and mean closest approach to airguns for all (untruncated) visual detection records. The most frequently sighted species were the sperm whale and pantropical spotted dolphin (sighting rates of 0.48 and 0.41 visual detections per 100 hours of observer effort, respectively).

**Table 7:** Summary of visual observations of cetaceans, excluding unidentified species, during seismic mitigation surveys in the Gulf of Mexico 2002–2015.

Species	Common Name	Number of Visual Detection Records	Mean Group Size	Mean Water Depth (m) of Detection Records	Sighting Frequency (per 100 hours of visual effort)	Mean CPA to Airguns^1^ (m)
*Balaenoptera ricei*	Rice’s whale^2^	38	1.3	989	0.006	1,684
*Kogia breviceps or sima*	Pygmy or Dwarf Sperm Whale	45	2.6	1,548	0.008	812
*Physeter macrocephalus*	Sperm Whale	2,898	2.2	1,452	0.484	1,732
*Ziphius spp*., *Mesoplodon spp*.	Beaked Whale	44	2.2	1,390	0.074	2,090
*Feresa attuata*	Pygmy Killer Whale	110	9.3	1,097	0.018	632
*Globicephala macrorhynchus*	Short-finned Pilot Whale	493	12.5	1,279	0.082	704
*Grampus griseus*	Risso’s Dolphin	141	8.2	1,194	0.024	698
*Lagenodelphis hosei*	Fraser’s Dolphin	69	32.8	1,550	0.012	917
*Orcinus orca*	Killer Whale	10	5.4	1,276	0.002	892
*Peponocephala electra*	Melon-headed Whale	156	29.3	1,249	0.026	728
*Pseudorca crassidens*	False Killer Whale	110	9.3	1,332	0.018	963
*Stenella attenuata*	Pantropical Spotted Dolphin	2,464	20.9	1,396	0.410	487
*Stenella clymene*	Clymene Dolphin	198	23.9	1,500	0.033	627
*Stenella coerulealba*	Striped Dolphin	36	27.9	1,732	0.006	1,247
*Stenella frontalis*	Atlantic Spotted Dolphin	388	14.6	222	0.060	193
*Stenella longirostris*	Spinner Dolphin	220	25.5	1,388	0.037	920
*Steno bredanensis*	Rough-toothed Dolphin	294	17.6	1,362	0.049	413
*Tursiops truncatus*	Bottlenose Dolphin	2,799	7.8	248	0.470	313

CPA = Closest Point of Approach.

^1^ –untruncated sighting distance includes all airgun operating levels.

^2^ –All reported baleen whales were classified as Rice’s whales for analysis.

Accuracy of species identifications did not always concur with a “positive” certainty of identification. While a PSO may be confident in their identification, it could still be incorrect. For example, in the raw PSO dataset used for the analysis detailed in this manuscript, eight records of common dolphin (*Delphinus delphinus*), two records of Atlantic white-sided dolphin (*Lagenorhynchus acutus)*, and one record of a long-finned pilot whale (*Globicephala melas*) were assigned a “positive” identification confidence code by PSOs. All these species are extralimital to the northern GOM making an encounter extremely rare and highly unlikely [8, 9]. Despite some of these potential identification errors, species records were overwhelmingly in line with what is known about species stocks in the GOM [8, 9]. Common species like pantropical spotted dolphins and sperm whales typically have a high percentage of “positive” identification confidence codes, whereas less common species like beaked whales show a higher percentage of “possible” and “probable” identification confidence codes. All species identifications for any confidence codes were accepted for species expected in the GOM.

Each NTL category (i.e., “whale” or “dolphin”) was comprised of various species as identified by vessel-based PSOs on seismic surveys (Table 2); however, within each NTL category there were species more regularly classified by PSOs than other species. Within the whale NTL category, over 94% of the observations were classified by PSOs as sperm whales. Within the dolphin NTL category over 50% of the observations were classified as either bottlenose dolphins or pantropical spotted dolphins.

Each auditory category was likewise generally dominated by a few individual species (Table 3). The low frequency group was presumed to consist only of the Rice’s whale (100%) based on the distribution of that species compared to other mysticetes in the GOM. Although a small percentage of other, more extralimital whale species could have been included in the Sighting Reports for this group, all would still be in the low-frequency group. The mid-frequency group was dominated by sperm whales (20%), bottlenose dolphins (22%), and pantropical spotted dolphins (25%). The high frequency group consisted entirely of *Kogia* spp. (100%).

### Closest point of approach (CPA)

Noise exposure studies aim to determine the level of behavioral responses to a noise stimulus and often include acoustic tagging of species to better quantify dive and sound exposure patterns [26, 27]. Behavioral reactions are highly complex and may not necessarily be identifiable, particularly from the perspective of a shipboard PSO [26–28]. PSOs do not typically have the expertise, tools, or survey conditions to fully and systematically assess behavioral reactions of species specifically to airgun operation in an objective manner [28, 29]. While behavioral descriptions during visual observations are often provided by PSOs in good detail, the lack of a defined classification framework [30] under the regulatory training and reporting requirements makes these observations largely subjective and unquantifiable. Therefore, CPA distances are used as a quantitative measure of potential behavioral avoidance. CPA distances to a noise stimulus have frequently been used as a metric to assess marine mammal behavior responses [13, 31–36]. However, an animal’s CPA to the airgun array may be influenced by sensitivity to airgun noise impulses, vessel presence, animal activity at the time of encounter, and other contextual criteria [16, 37, 38]. Analysis of behavioral reactions are further complicated by potential observer bias, variability in survey configuration, expertise of PSOs, and accuracy of reporting [13, 36]. Therefore, CPA distances may only be one, albeit important, component of response analyses; the full assessment of complex behaviors requires other research beyond that which could be expected to emerge from regulatory mitigation program reporting.

The number of CPA observations per airgun power level for each NTL group is summarized in Table 8. For all NTL categories with observations truncated to within 2,500 m of airgun arrays most observations (>62%) occurred during full power activities, with the fewest observations (<3%) made during ramp up (Table 8). This breakdown was similar for NTL categories truncated to within 500 m of airgun arrays. For this truncation distance most observations (>58%) were made during full power activities, while the fewest observations (<3%) were made during ramp up (Table 8). Airgun operations were not equally represented across airgun power levels and detection rates and therefore affected the number of observations at each level. Data available in the PSO reports for recording airgun operations and effort at each power level were poorly recorded and highly inconsistent and could not be quantified by the total amount of time for each power level. However, in general, based on review of the total visual effort data, most effort was recorded at full power operations, followed by silent and the least amount of effort recorded for ramp up.

**Table 8:** Total number (n) and percentage (%) of closest point of approach observations per airgun activity level for each data truncation distance (m) and NTL category examined.

Truncation Distance (m)	NTL Category	Total Observations (n)	Full Power		Minimum Source		Ramp up		Silent	
			n	%	n	%	n	%	n	%
2,500	All Whale Species	2,291	1,511	66.0	153	6.7	48	2.1	579	25.3
	All Dolphin Species	8,338	5,245	62.9	638	7.7	192	2.3	2,263	27.1
500	All Whale Species	--	--	--	--	--	--	--	--	--
	All Dolphin Species	5,524	3,240	58.7	453	8.2	123	2.2	1,708	30.9

-- = data not applicable.

The number of CPA observations per airgun activity level for each auditory group is shown in Table 9. Most observations pertaining to each auditory group were made during full power activities (>52%), with the fewest made during ramp up (<4%). In the low- and high-frequency auditory categories there were less than three observations each for minimum source and ramp up activity levels (Table 9). Due to the very low number of observations for these categories, they were excluded from the statistical analysis. All airgun activity levels were therefore investigated only for the mid-frequency auditory group.

**Table 9:** Total number (n) and percentage (%) of closest point of approach observations per airgun activity level and auditory category examined^1^.

Auditory Group	Total Observations (n)	Full Power		Minimum Source		Ramp up		Silent	
		n	%	n	%	n	%	n	%
Low Frequency (7 Hz to 35 kHz)	25	14	56.0	3	12.0	1	4.0	7	28.0
Mid Frequency (150 Hz to 160 kHz)	10,521	6,697	63.7	785	7.5	235	2.2	2,804	26.7
High Frequency (275 Hz to 160 kHz)	40	21	52.5	2	5	0	0.0	16	40.0

^1^Data represents truncation to include only those observations within 2,500 m of airgun arrays during seismic surveys.

#### Whales

Table 10 summarizes results for all observed CPA comparisons conducted for whales. There was an overall statistical difference in the mean CPA per airgun activity level for combined whale species (F_3,2287_ = 6.3; P_α = 0.05_ = 0.0003), with whales being significantly further from airgun arrays during full power when compared to silent (t_2287_ = 4.1; P_α = 0.05_ = 0.0002). Sperm whales (t_2143_ = 14.3; P_α = 0.05_ = 0.002) and *Kogia* species (F_1,36_ = 4.51; P_α = 0.05_ = 0.04), had observed CPAs significantly further at full airgun activity level than silent.

**Table 10:** Summary of observed whale CPA distances that were greater or less when comparing each airgun activity level.

All Whales	Sperm Whales	Baleen Whales	*Kogia* spp.	Beaked Whales
*Full > Silent	*Full > Silent	Full > Silent	*Full > Silent	Full > Silent
Full > Minimum Source	Full > Minimum Source	Full < Minimum Source	Full > Minimum Source	Full > Minimum Source
Full < Ramp up	Full < Ramp up	Full > Ramp up	N/A	N/A
Minimum Source < Silent	Minimum Source > Silent	Minimum Source < Silent	Minimum Source < Silent	Minimum Source < Silent
Ramp up > Silent	Ramp up > Silent	Ramp up > Silent	N/A	N/A
Minimum Source < Ramp up	Minimum Source < Ramp up	Minimum Source < Ramp up	N/A	N/A

*Denotes comparisons that are statistically significant.

#### Dolphins

There was an overall statistical difference in mean observed CPA per airgun activity for combined all dolphin species (F_3,8334_ = 70.5; P_α = 0.05_ <0.0001) with dolphins observed significant farther during full power than silent (t_8334_ = 14.5; P_α = 0.05_ <0.0001) and minimum source power (t_8334_ = 3.2; P_α = 0.05_ = 0.007). Additionally, observed dolphin CPAs were significantly farther during ramp up than silent (t_8334_ = 3.4; P_α = 0.05_ = 0.005). Back transformed mean, and raw mean for observed dolphin CPA distances are provided in Table 11.

**Table 11:** Back transformed mean, and raw mean (± standard deviation [SD]) distance (m) of all dolphin species closest point of approach observations within 2,500 m of seismic survey airgun arrays.

Airgun Activity Level	Back Transformed Mean	Statistical Difference	Raw Mean (± SD)
Full	411	a	542 ± 566
Minimum Source	352	b	459 ± 487
Ramp up	361	ab	482 ± 527
Silent	261	c	382 ± 493

Different letters denote statistical difference of back transformed means at P ≤0.05.

Table 12 summarizes results for all observed CPA analyses conducted for dolphin data truncated at 2,500m. For all dolphin species observed within 500 m of airgun arrays during seismic surveys there was also an overall statistical difference in the mean CPA per airgun activity level (F_3,5520_ = 27.3; P_α = 0.05_ <0.0001) with observed dolphin CPA distances significantly further during minimum source compared to silent (t_5520_ = 7.0; P_α = 0.05_ <0.0001); significantly further during full compared to minimum source power (t_5520_ = 2.8; P_α = 0.05_ = 0.03) and silent activities (t_5520_ = 7.8; P_α = 0.05_ <0.0001); and significantly further during minimum source compared to ramp up (t_5520_ = 2.8; P_α = 0.05_ = 0.03).

**Table 12:** Summary of observed dolphin CPA distances that were greater or less when comparing each airgun activity level.

All Dolphins	Bottlenose & Pantropical Spotted Dolphins	Other small dolphins^1^	Blackfish^2^
*Full > Silent	*Full > Silent	*Full > Silent	*Full > Silent
*Full > Minimum Source	*Full > Minimum Source	*Full > Minimum Source	Full > Minimum Source
Full > Ramp up	*Full > Ramp up	Full > Ramp-up	*Ramp up> Full
*Minimum Source > Silent	*Minimum Source > Silent	*Silent > Minimum Source	*Minimum Source > Silent
*Ramp up > Silent	Silent > Ramp up	Silent > Ramp up	*Ramp-up >Silent
Ramp up > Minimum Source	*Minimum Source > Ramp up	Minimum Source > Ramp up	*Ramp up > Minimum Source

*Denotes comparisons that are statistically significant.

^1^Bow riding dolphins are a subset of dolphins defined by data collected after 2014 that confirmed bow riding behavior as part of the sighting detection report

^2^Blackfish are defined as members of the *Delphindae* family characterized by medium to large sized dolphins that are predominately dark in color and non-descript when viewed at a distance. Species within this non-taxonomic group include false killer whales, killer whales, melon-headed whales, pilot whales, and pygmy killer whales.

The CPA results in this study are similar to those found by Stone and Tasker [33] where small odontocetes, killer whales, and mysticetes all remained significantly farther from airguns operating (power levels not defined) than silent airguns. In contrast, our results showed that sperm whales were seen significantly farther from airgun during full power versus silent airguns with no such significant difference reported. Given that sperm whales drove much of our data, this distance was significant for the *All Whales* category. Analysis of 12 years of JNCC seismic mitigation data showed similar results for all baleen whales, killer whales, three species of bow-riding dolphins, and harbor porpoise, with significantly farther distances from active “large arrays” versus silent [13].

#### Auditory groups

Airgun modeling shows that the majority of airgun source energy is contained below 1 kHz [1]. Because behavioral reactions may be based on hearing sensitivities, analysis of CPA distances by each of the hearing groups was conducted. For low-frequency and high-frequency species, only full power and silent comparisons could be made due to the low number of species detections within those groups.

*Low-frequency auditory group (7 Hz—35 kHz)*. The low frequency auditory group consisted solely of Rice’s whales for which no statistical difference (F_1,19_ = 1.0; P_α = 0.05_ = 0.32) in the mean observed CPAs was observed between full and silent airgun activity levels (Table 10). Comparisons of airgun activity levels against minimum source and ramp up activities were not possible due to the very low number of observations for these categories (Table 3).

*Mid-frequency auditory group (150 Hz—160 kHz)*. There was an overall statistical difference in the transformed mean CPA per airgun activity level of all mid-frequency cetaceans observed within 2,500 m of airgun arrays during seismic surveys (F_3,10518_ = 69.4; P_α = 0.05_ <0.0001). Mid-frequency cetaceans had observed CPA distances that were significantly further during full power than silent (t_10518_ = 14.3; P_α = 0.05_ <0.0001); during minimum source power than silent (t_10518_ = 4.1; P_α = 0.05_ = 0.0002), during ramp up activities than silent (t_10518_ = 4.1; P_α = 0.05_ = 0.003); and during full power than minimum source (t_10518_ = 4.1; P_α = 0.05_ = 0.0002). Back transformed mean, and raw mean for observed mid-frequency group CPA distances are provided in Table 13.

**Table 13:** Back transformed mean, and raw mean (± standard deviation [SD]) distance (m) of mid-frequency cetaceans closest point of approach observations within 2,500 m of seismic survey airgun arrays.

Airgun Activity Level	Back Transformed Mean	Statistical Difference	Raw Mean (± SD)
Full	542	a	700 ± 665
Minimum Source	455	b	594 ± 602
Ramp up	465	ab	615 ± 634
Silent	371	c	529 ± 611

Different letters denote statistical difference of back transformed means at P ≤0.05.

The mid-frequency auditory group encompasses a highly diverse group of cetaceans and includes those species that are most commonly observed by PSOs during seismic surveys (Table 3). The analysis on this large and diverse grouping has the potential to mask species-specific responses to various airgun activity levels. Therefore, to better determine how airgun activity levels affect mid-frequency cetaceans that have differentiated autecological characteristics, this group was broken down into five subcategories which were each analyzed separately (Table 14).

**Table 14:** Number of observations of each mid-frequency faunal group per airgun activity level.

Faunal Grouping	Number of Observations (n)				Total Percentage (%) of Mid-Frequency Observations
	Full	Minimum Source	Ramp up	Silent	
Sperm Whale	1,433	145	43	526	20.4%
Bottlenose and Pantropical Spotted Dolphins	3,966	546	113	271	46.5%
Other Small Dolphins	644	38	6	413	10.5%
Blackfish	416	32	67	444	9.1%
Beaked Whales	17	2	0	15	0.3%

***Sperm whales*.** Sperm whale observations make-up 20.4% of the total number of mid-frequency auditory observations (Table 12). There was an overall statistical difference in the mean CPA per airgun activity level for sperm whales observed within 2,500 m of airgun arrays during seismic surveys (F_3,2143_ = 4.75; P_α = 0.05_ = 0.003) with observed CPA distances significantly farther during full power than silent (t_2143_ = 14.3; P_α = 0.05_ = 0.002). There were no statistically significant differences in observed CPA distance between other airgun activity comparisons for sperm whales.

#### Bottlenose and pantropical spotted dolphins / other small dolphins

The majority of observations (47%) made within the mid-frequency auditory grouping were of bottlenose and pantropical spotted dolphins. There was an overall statistical difference in the transformed mean CPA per airgun activity level (F_3,4892_ = 62.51; P_α = 0.05_ <0.0001). Observed CPA distances were significantly farther during full power than silent (t_4892_ = 11.31; P_α = 0.05_ <0.0001), minimum source (t_4892_ = 4.44; P_α = 0.05_ <0.0001) or ramp up (t_4892_ = 7.56; P_α = 0.05_ <0.0001); and significantly further during minimum source than silent (t_4892_ = 6.82; P_α = 0.05_ <0.0001) or ramp up (t_4892_ = 5.02; P_α = 0.05_ <0.0001). Back transformed mean, and raw mean for observed mid-frequency group CPA distances are provided in Table 15.

**Table 15:** Back transformed mean, and raw mean (± standard deviation [SD]) distance (m) of the bottlenose dolphin and pantropical dolphin closest point of approach observations within 2,500 m of seismic survey airgun arrays.

Airgun Activity Level	Back Transformed Mean	Statistical Difference	Raw Mean (± SD)
Full	413	a	537 ± 565
Minimum Source	329	b	432 ± 467
Ramp up	157	c	202 ± 248
Silent	160	c	232 ± 309

Different letters denote statistical difference of back transformed means at P ≤0.05.

Analysis of CPAs for all other small dolphins that were identified to species showed similar results to the pantropical spotted dolphin and bottlenose dolphin combined results. There was an overall statistical difference in the transformed mean CPA per airgun activity level (F_2,1092_ = 60.34; P_α = 0.05_ <0.0001) with observed CPA distances for these dolphin species significantly further away during full power compared to silent (t_1092_ = 10.07; P_α = 0.05_ <0.0001) and during minimum source power compared silent (t_1092_ = 6.55; P_α = 0.05_ <0.0001). In contrast to the bottlenose/pantropical group, the other dolphin group had observed CPAs that were significantly farther during silent than during minimum source levels (t_1092_ = 2.85; P_α = 0.05_ <0.012).

#### Blackfish

Blackfish are defined as members of the *Delphindae* family comprising medium to large sized dolphins that are predominately dark in color and non-descript when viewed at a distance. Species within this non-taxonomic group include false killer whales, killer whales, melon-headed whales, pilot whales, and pygmy killer whales. Blackfish species account for approximately 9% of the total observations within the mid-frequency auditory group (Table 14).

There was an overall statistical difference in the transformed mean CPA per airgun activity level (F_3,995_ = 353; P_α = 0.05_ <0.0001), but unlike most other mid-frequency and dolphin groups, ramp up CPAs were comparatively farther from several airgun activity levels (Table 12). Blackfish had observed CPA distances significantly further during ramp up compared to full (t_955_ = 5.36; P_α = 0.05_ <0.0001), minimum source activities (t_955_ = 4.14; P_α = 0.05_ = 0.0002), and silent (t_955_ = 20.63; P_α = 0.05_ <0.0001). observed CPA distances were significantly further during full compared to silent (t_955_ = 29.28; P_α = 0.05_ <0.0001) and during minimum source compared to silent (t_955_ = 9.91; P_α = 0.05_ <0.0001). Back transformed mean, and raw mean for observed mid-frequency group CPA distances are provided in Table 16.

**Table 16:** Back transformed mean, and raw mean (± standard deviation [SD]) distance (m) of the blackfish faunal grouping’s closest point of approach observations within 2,500 m of seismic survey airgun arrays.

Airgun Activity Level	Back Transformed Mean	Statistical Difference	Raw Mean (± SD)
Full	550	b	615 ± 417
Minimum Source	485	b	585 ± 527
Ramp up	838	a	898 ± 528
Silent	62	c	115 ± 289

Different letters denote statistical difference of back transformed means at P ≤0.05.

#### Beaked whales

Beaked whales comprised less than 1% of all mid-frequency group observations (Table 14). There was no statistical difference (F_1,30_ = 0.78; P_α = 0.05_ = 0.39) in the mean observed CPA of beaked whales during full and silent activities within 2,500 m of airgun arrays. Comparisons of airgun activity levels against minimum source and ramp up activities were not possible because there were no observations of these species during ramp up activities and only two observations during minimum source power.

*High frequency auditory group (275 Hz—160 kHz)*. Only one genus (*Kogia*) is represented in the high frequency group. The observed CPA distances for *Kogia* during full power were significantly further compared to silent (F_1,36_ = 4.5; P_α = 0.05_ = 0.04). These high frequency cetaceans were generally observed to be approximately 600 m further away from airgun arrays during full power when compared with silent activities. Comparisons of airgun activity levels against minimum source and ramp up activities were not possible due to the very low number of observations for these categories.

### Effectiveness of ramp up and minimum source as mitigation

CPA was used as a very generalized determination of ramp up and minimal source mitigation effectiveness based on animal ranges to varying source operational levels. The purpose for operating airguns at the minimum source level or implementing a ramp up is to use a lower source output level to “warn” animals away from an ensonified area before the source output reaches full power. In theory, animals should be farther away, or moving away, from an active array during ramp up or minimal source operations than at silence. Ramp up as a mitigation practice, starts from silence, therefore comparisons of CPA collected during ramp up versus full power may not be as applicable as comparisons of ramp up with silent. Evaluating an animal’s relative position and bearing, swim speed and behavior at the start of ramp up to the same parameters at the end of ramp up would provide an indication of whether animals are moving away. Sperm whale and Rice’s whale swim direction during full power and silence were summarized from PSO reports that contained enough information to place swim direction into one of 5 categories: 1) towards the vessel; 2) away from the vessel; 3) parallel to the vessel in the same direction as vessel; 4) parallel to the vessel in the opposite direction as vessel; and 5) crossing perpendicular either in front of or behind the vessel. Notably, ramp up airgun activity was not assessed mainly because ramp ups would be delayed or shut down with a whale within 500m of the arrays; and therefore, the variability introduced by that mitigation measure, combined with low numbers for that specific scenario, is inconsistent for assessment of a full ramp up.

A chi-square test of independence was performed on 1,686 whale detection records to examine the relationship between airgun operation level (i.e., full and silent) and whale travel behavior in relation to the vessel. The relationship among these variables was not significant, X22,1686 = 5.30, P α = 0.05 = 0.07. This finding indicates that the observed and expected frequencies were generally similar to each other and no clear correlation between airgun activity level and swim direction could be presumed.

Overall, ramp up effectiveness in regard to reducing an animal’s acoustic exposure based on adjustment in behavior or movement has not been fully evaluated. Controlled studies have shown mixed results even though it is a widespread mitigation method in many at-sea operations and its effectiveness is of particular importance to regulators. Wensveen [39] tested ramp up effectiveness during sonar surveys by modeling the predicted sound exposure received by several tagged odontocete species. Animals first exposed to a ramp up of the military sonar displayed aversion behavior that subsequently resulted in reducing their predicted acoustic exposure within the modeled sound fields; however, in subsequent ramp ups their movement and behavior did not result in significantly reducing their predicted risk to acoustic exposure suggesting that the animals may become habituated to ramp ups [39]. Additionally, Wensveen [39] based assessment of avoidance behavior on changes in animal headings. Comparable data that are reliable in PSO reports do not exist because when headings are recorded accurately, they are only given for the initial detection with subsequent heading changes not recorded. Although Dunlop [35] reported a general response by humpback whales to move away from an airgun source, there was no evidence that ramp up methodology triggered the avoidance response versus a constant source. Stone [13] found variable results when comparing “toward vessel” versus “away from vessel” behavior during ramp up versus other airgun operations. Although Stone [13] reported a general trend for travel “away from the vessel” during ramp up although, results were mixed with no significant differences. PSO data summarized from the corresponding BOEM studies report [10] show similar variable results. Qualitative PSO descriptions suggest a greater percentage of dolphin records traveling toward versus away from the vessel and more whales were recorded by PSOs as moving “away” versus “toward” the vessel during ramp up [10].

Minimum source operation has a different premise than ramp up, in that it is used after airguns have reached full power so that, theoretically, the ensonified field will keep animals away from the highest noise exposures. In this regard, mitigation effectiveness could be indicated by CPA distances equal to or greater than those observed at full power, or CPAs at minimum source power that are greater than those at the silent stage.

For whales, there was no statistical difference in CPA distances between full power operations and minimum source or ramp up operations. This suggests that whales occur at similar distances from the operating sources / vessels regardless of specific airgun array output levels. There was also no significant difference in the CPA between airguns that were silent, and airguns operating at minimum source or ramp up; this indicates that ramp up or minimum source levels may not fulfill the intended consequences of avoidance. However, without behavioral and movement information it is difficult to fully assess the effectiveness of these mitigation measures.

Dolphins showed significant differences in CPA distances between minimum source levels and all other airgun activities, but no significant differences between ramp up and other airgun activities. Minimum source operations are problematic to evaluate because a standard method is not used and is equipment-dependent, meaning minimal sound pressure levels could range from the NTL-specified 160 dB re 1 μPa to nearly full power operations. Additionally, timing of the minimum source firing is supposed to operate at the same shot point interval as the survey array; however, there are no records for confirming this procedure (i.e., no record of the actual source output level or shot point intervals when at minimum power by which to gauge the propagated sound levels at different distances). Therefore, variability in the source application itself as well as potential variability in what the animals perceive may affect the efficacy of minimal sourcing as a mitigation method. The effectiveness of ramp up and minimum source operations are likely dependent upon individual species and the context of the received levels. Without a full understanding, and measurements, of propagated signals levels, ambient noise conditions, and changing environmental soundscapes, it is unclear whether there is a received level threshold that is tolerated by the animals or whether whales and dolphins are responding to minimum source or ramp up activities.

### Other mitigation

While not related to CPA analysis, mitigation requirements included operational shutdowns for whales (Table 2); and ramp up delays for whales and dolphins. A shut down is defined as turning off any airgun activity that is in progress including ramp up, minimal source power, or full power at the time a whale enters the shutdown zone (500 m). A ramp up delay results when a whale or dolphin is within the 500-m clearance zone immediately prior to initiating airgun activity. PSOs implemented a total of 260 operational shutdowns from visual detections of a whale inside or entering the 500-m shut down zone. Shut downs were initiated 248 times for sperm whales, 2 for baleen whales (assumed to be Rice’s whales), 6 for *Kogia* spp, 2 for beaked whales, and 2 for unidentified whales. The mean duration of all shutdowns was 63 minutes which includes a required 30-minute post shut down clearance period. PSOs visually initiated 39 delays for whales comprising roughly the same species proportions as shutdowns, and 287 delays for dolphin species. The mean duration of ramp up delays from visual detections was 22 minutes for whales and 40 minutes for dolphins.

## Conclusions and recommendations

This paper endeavored to provide insight for applying PSO data collected during seismic surveys to the broader knowledge base regarding marine mammals and potential effects on behavior from seismic surveys. The results are specific to the GOM and the predominant odontocete population that are found on the OCS. There were data quality issues mainly due to simple input and submission errors and inconsistencies, and not due to poor mitigation and monitoring conducted by the PSOs. Despite the quality issues, the PSO reports provided a robust and unequalled dataset for GOM that can be used for ongoing research, assessment, management and regulatory planning. Based on data collected by PSOs during 2002–2015 seismic surveys in the GOM, nearly all species groupings were observed farther away from active versus silent airguns, indicating a potential for avoidance behavior similar to those documented in other studies for seismic surveys [15, 31, 32, 34, 35] and other anthropogenic sources [39–42].

For whales, the observed CPA to airgun arrays was significantly further away (by 140 m) from airguns operating at full power versus silent. However, this result was driven largely by the large proportion of sperm whales for which mean CPA distances 127 m farther at full power than at silence indicating that sperm whales may position farther from airguns operating at full power verses airguns at silence.

Similarly, for dolphins, observed CPA distance was significantly farther for all power levels combined versus silent airguns. This suggests a potential behavioral response to any active source when compared to airgun silence for dolphins. However, given that dolphin bow ride and have the closest observed CPAs to full power arrays compared to other groups, there are likely other factors influencing movements.

For the one low-frequency species, Rice’s whale, no significant differences in CPA distances were evident between full power and silent based on the limited sample size (n = 25). However, among high-frequency species, CPA distances were significantly farther during full power versus silent.

Unlike the combined mid-frequency group, beaked whales showed no significant difference between CPAs during full power versus silent airgun operations. However, as a group, they have shown particular sensitivity to disturbance [42, 43] and higher frequency auditory sensitivities and vocalizations when compared to the broader mid-frequency group [36, 41]. In comparison to all other groups analyzed, beaked whales have the largest mean CPA distance (2,090 m) for all airgun activities combined. Blackfish showed clear and significant differences (584 m farther) for CPAs during any airgun operations versus silence. Blackfish also showed marked differences between CPAs during ramp up and other airgun operation levels. Mitigation actions are typically applied equally by regulatory bodies to all cetaceans in the family Delphinidae; given the marked difference we found in CPA distances; further investigation may be warranted to address the potential sensitivity of blackfish species within the mitigation framework.

Mitigation measures can only be effective if they can be practically applied in real-world situations; therefore, personnel, equipment, and safety requirements must all be considered for any proposed mitigation measures. Since 2002, the seismic industry has borne the burden of additional personnel and equipment on board vessels and has demonstrated a high level of compliance with required mitigation measures and self-reporting requirements, and has supported initiatives for improving PSO efficacy, standards, and data collection. Mitigation measures should evolve with the science of impact assessment and efficacy evaluation. Although monitoring methods and mitigation actions necessarily begin on intuitive measures, such measures should continue to evolve through adaptive management based on science-based feedback on the efficacy of these efforts.

As is often the case with complex data sets, our analysis identified opportunities for improvement and further examination. During the initial data review process, it became clear that the PSO data examined could benefit from systematic and ongoing QAQC to ensure consistency and standardization. Improving this process would better facilitate evaluating the efficacy of mitigation and monitoring methods and the utility of PSO data for understanding species occurrence, distributions, and behavior. For example, our analyses indicate current processes can benefit from enhanced spatial and effort tracking; measurement of sound sources and ambient soundscapes to better understand acoustic characteristics of exclusion zones, ramp up propagation, and minimal source sound fields; and animal exposures within each of those scenarios.

PSO data collection is critical to long-term management of protected species and industry requirements in the GOM. As questions regarding the data evolve, transmission of needs and how to statistically address those needs should be a priority for mitigation data collection going forward. Recommendations to improve the utility of data collected by PSOs include:

- Robust data collection training including immediately prior to each PSO deployment and PSO “refresher” courses required at regularly intervals;
- Develop and implement PSO data collection standards (e.g., templates, data dictionaries) across all OCS activities and regions.
- Use of a standardized electronic data collection template and centralized electronic data submission repository that will immediately identify incorrectly entered data. Correction of incorrect data should then be required for permit compliance;
- Develop protocols for reporting detections on multi-vessel surveys to resolve duplicative sightings, without compromising individual vessel compliance;
- Require accurate range and bearing information to each sighting and vessel position information, to allow calculation of animal location rather than recording the animal at the same location as the vessel;
- Develop and implement standardized behavior code definition appropriate for mitigation observers;
- Improve observer effort recording that aligns with scientific surveys so that observation bias can be assessed; and
- Require use of location-calibrated reticle binoculars or similar devices for distance estimation.
- As more technology becomes available the input and submission processes will hopefully improve; however as novel methods go online, the same basic principles discussed above should be equally, if not more stringently, applied to ensure proper evaluation of these technologies.

One of the cornerstone authorities related to Federal information collection is the Paperwork Reduction Act (PRA) of 1980 [44 U.S.C. 3501 et seq.] as amended in 1986 and 1995. The PRA was passed to minimize the Federal paperwork burden on the public and improve the quality and use of Federal information. Under the PRA, Federal Agencies must obtain approval from the Office of Management and Budget (OMB) before undertaking information collection directed at ten or more persons. Importantly, PSO reporting is part of a larger mitigation effort designed to minimize acoustic impacts to marine mammals and sea turtles. Before enacting large-scale change through the PRA, it seems prudent that data improvement should start within the PSO personnel community. This in turn suggests consideration of more consistent training and standards which are now being implemented through the regulatory incidental take authorization process (88 Federal Register [FR] 916). Similar to the American National Standards Institute (ANSI) PAM standards working group [15], we recommended that similar standards language and reporting requirements be developed for PSO surveys across the seismic industry.
